# Thyroid Carcinoma with Pituitary Metastases: 2 Case Reports and Literature Review

**DOI:** 10.1155/2015/252157

**Published:** 2015-01-21

**Authors:** Weiying Lim, Dawn Shaoting Lim, Chiaw Ling Chng, Adoree Yiying Lim

**Affiliations:** Singapore General Hospital, Outram Road, Singapore 169608

## Abstract

We present 2 patients with pituitary metastases from thyroid carcinoma—the first from anaplastic thyroid carcinoma and the second from follicular thyroid carcinoma. The first patient, a 50-year-old lady, presented with 2-week history of hoarseness of voice, dysphagia, dyspnoea, and neck swelling. Imaging revealed metastatic thyroid cancer to lymph nodes and bone. Histology from surgery confirmed anaplastic thyroid cancer. She was found to have pituitary metastases postoperatively when she presented with nonvertiginous dizziness. She subsequently underwent radiotherapy and radioiodine treatment but passed away from complications. The second patient, a 65-year-old lady, presented with loss of appetite and weight with increased goitre size and dyspnoea. Surgery was performed in view of compressive symptoms and histology confirmed follicular thyroid carcinoma. Imaging revealed metastases to bone, lung, and pituitary. She also had panhypopituitarism with hyperprolactinemia and diabetes insipidus. She received radioiodine therapy but eventually passed away from complications.

## 1. Introduction

Pituitary metastases are rare. They are found in only 1% of all pituitary resections and only 2% of these are from thyroid carcinoma [1]. We report two unusual cases of anaplastic and follicular thyroid cancers who presented with pituitary metastases. A literature review of these rare metastases and the challenges encountered in the management of the pituitary metastases in these cases were also highlighted in this report [1, 2].

## 2. Case Presentation

### 2.1. Case 1

A 50-year-old woman presented with a 2-week history of progressive neck swelling, hoarseness of voice, dyspnoea, and dysphagia. Systemic review was otherwise unremarkable. Physical examination revealed a hard thyroid mass extending from the thyroid cartilage to just above the supraclavicular notch. Initial fine needle aspiration cytology (FNAC) of the thyroid, done at another institution, was suspicious for Hurtle cell carcinoma.

**Figure 1:**
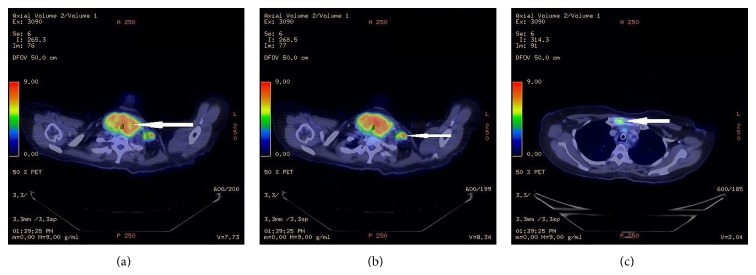
PET-CT scan showing (a) hypermetabolic enlarged thyroid gland, (b) hypermetabolic left cervical lymph node, and (c) hypermetabolic focus in right side of manubrium (indicated by arrows).

Three months later, she sought a second opinion at our institution. Computed tomography (CT) and F-18-FDG-positron emission tomography (PET) whole body scans (from skull vertex to upper thighs) were done to evaluate the extent of disease and revealed aggressive thyroid carcinoma with metastases to the manubrium and left cervical levels II–IV and supraclavicular lymph nodes (Figures 1(a)–1(c)). She underwent a total thyroidectomy, laryngectomy with bilateral neck dissection 3 weeks later. Histology revealed poorly differentiated thyroid carcinoma of TNM stage T4N1M1. The tumor measured 6.5 cm × 5.5 cm × 2.3 cm with large area of central necrosis and extrathyroidal extension to the perithyroidal soft tissue and laryngeal cartilage. 78 cervical lymph nodes were examined and 8 were involved (10%). BRAF mutation was negative.

She was discharged but readmitted 26 days after surgery with nonvertiginous dizziness. Magnetic resonance imaging (MRI) of the brain showed enhancing tissue in the left cavernous sinus, left V3 branch of trigeminal nerve, and left sphenoid sinus. There was also involvement of the pituitary gland and erosion of the left lateral aspect of sellar floor. Hormonal profile showed normal pituitary function and no evidence of diabetes insipidus. She was scheduled for 10 fractions of 30 Gy palliative radiotherapy to the base of skull and cavernous sinus metastasis. Her postoperative thyroglobulin level was 704 UG/L (2–70 UG/L), with negative thyroglobulin antibodies.

**Figure 2:**
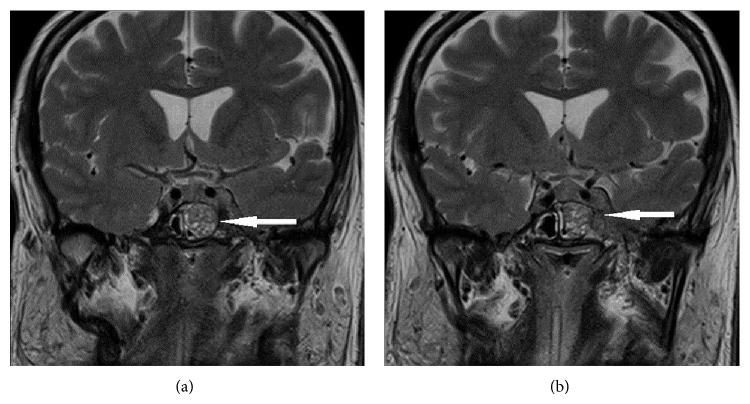
MRI brain (T2-weighted, contrast, and coronal) showing (a) pituitary involvement and (b) invasion into left cavernous sinus (indicated by arrows).

However, she developed left ptosis and diplopia while undergoing radiotherapy. Physical examination revealed left complete ptosis and incomplete left eye adduction. Repeat MRI imaging of the pituitary gland showed enlargement of the pituitary gland with mildly thickened infundibular stalk and a stable left cavernous sinus mass measuring 25 × 27 × 22 mm with encasement of the cavernous portion of the left internal carotid artery (Figures 2(a) and 2(b)).

She completed the full course of radiotherapy and was also given a single ablative dose of radioactive iodine at 200 mci. The pituitary metastasis was non-iodine-avid. Unfortunately, she showed no improvement in her clinical condition and passed away a few months later.

### 2.2. Case 2

A 65-year-old woman presented with loss of appetite and weight, increased goitre size, and increased dyspnoea. Systemic review was unremarkable. Physical examination revealed a nodular goitre with retrosternal extension with no associated cervical lymphadenopathy.

CT scan revealed a large L4 vertebral body lytic lesion and an 8 mm pulmonary nodule in the left lung base, suspicious for metastases. She underwent a CT-guided biopsy of the L4 vertebral body lytic lesion which revealed the presence of tumour cells which stained positive for CK7, TTF-1, and thyroglobulin. These findings suggested possible primary pulmonary or thyroid malignancy. FNAC of the thyroid, however, revealed benign follicular cells with focal Hurtle cell changes.

CT scan of the neck was performed to evaluate the degree of airway obstruction and, limited views of the brain, incidentally revealed a pituitary mass. There was asymmetric enlargement of the thyroid with heterogeneous enhancement and areas of calcifications. The left thyroid lobe measures 6.7 × 5.8 × 8.4 cm while the right thyroid lobe measures 3.5 × 3.9 × 6.2 cm. There is mass effect with deviation of the trachea to the right side. At its narrowest point, the trachea measures approximately 0.6 × 1.3 cm. No significant cervical lymphadenopathy was noted.

The pituitary mass was further evaluated by MRI imaging. It measured 2.5 × 2.7 × 2.2 cm with some haemorrhagic changes and invasion of the left cavernous sinus. Mass effect noted on the optic chiasm on imaging prompted formal ophthalmological examination, which showed right central scotoma with left inferior hemifield defect. Her hormonal profile revealed panhypopituitarism, hyperprolactinemia from “stalk effect,” and diabetes insipidus (Table 1).

Given the clinical context of an enlarging goitre with relative rapidity, the likelihood of a thyroid malignancy was deemed more probable than pulmonary malignancy. In view of significant obstructive symptoms, she underwent a total thyroidectomy without bilateral neck dissection and histology revealed a diagnosis of follicular thyroid carcinoma with capsular and vascular invasion of T4N0M1 staging. Test for BRAF mutation was not performed. Her postsurgical thyroglobulin was 11710 UG/L (2–70 UG/L) with a corresponding thyroglobulin antibody of 835 U/mL (0–60 U/mL).

**Figure 3:**
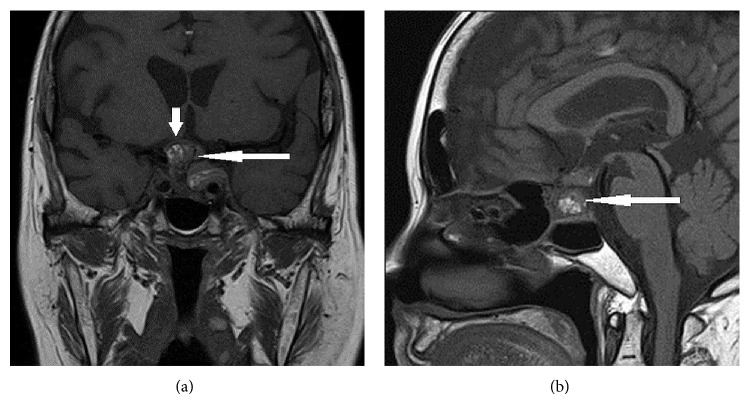
(a) MRI pituitary (T1-weighted, contrast, and coronal) showing pituitary mass (long arrow) with mass effect on the optic chiasm (short arrow). (b) MRI pituitary (T1-weighted, contrast, and sagittal) showing presence of increased signal intensity in the pituitary mass, representing presence of blood products (long arrow).

She had progressive blurring of vision and a repeat MRI pituitary scan showed a further enlargement of the pituitary mass, with dimensions of 3.2 × 3.0 × 1.9 cm and increased mass effect on the optic chiasm with extension into bilateral cavernous sinuses (Figures 3(a) and 3(b)).

She underwent a transsphenoidal resection of the pituitary mass which confirmed the presence of pituitary metastases from the thyroid follicular carcinoma. She subsequently suffered a left femur shaft fracture after a fall requiring internal fixation. Tissue histology from the fracture site revealed thyroid follicular carcinoma. She received 2 doses of radioactive iodine (250 mci each approximately 5 months apart) but unfortunately passed away from intraventricular haemorrhage after the second RAI treatment. The pituitary metastases were iodine-avid and after first RAI thyroglobulin was 27490 UG/L.

## 3. Discussion

Metastatic tumours can involve the pituitary by means of haematogenous spread, direct invasion from skull base metastasis, or meningeal spread through the suprasellar cistern [3]. Haematogenous spread can occur via the hypophyseal arteries or the portal system. As the posterior lobe of the pituitary is directly exposed to the arterial circulation, metastases to the pituitary gland from other malignancies have a predilection for the posterior lobe. However, pituitary metastases from thyroid carcinomas seem to differ from the norm, with diabetes insipidus affecting only 5 out of 22 of the reported cases (summarised in Table 2). One suggested reason for this phenomenon is that pituitary metastases from thyroid carcinomas tend to be relatively rapid growing parasellar lesions rather than intrasellar lesions that destroy pituitary tissue or interrupt the pituitary stalk [4], hence presenting more often with mass effect.

From the reported cases, direct invasions from skull base metastasis tend to be associated with large metastasis and are mostly from follicular thyroid carcinoma. In our first case, there was erosion of the sellar floor but it is difficult to ascertain the mode of spread in view of the rapid and aggressive growth of the carcinoma. In our second case, there was selective involvement of the pituitary gland with no evidence of bony involvement or meningeal enhancement; hence haematogenous spread is most likely.

Patients can present with symptoms related to mass effect from parasellar mass enlargement, such as ptosis, blurring of vision, and oculomotor and abducens nerve palsies. Rarer symptoms include diabetes insipidus and hypopituitarism. Our first case presented with oculomotor nerve palsy and preserved pituitary gland function. Our second case presented with panhypopituitarism, diabetes insipidus, and optic neuropathy. In the first case, the initial F-18-FDG-PET/CT scan did not reveal the pituitary metastasis. We postulate two reasons: (i) aggressive cancer (the pituitary metastasis was not present initially but developed quickly over the course of 3 months); (ii) F-18-FDG-PET scan does not pick up pituitary metastasis well [5] (the brain being very metabolically hyperactive would appear FDG-avid and may mask any pituitary metastasis).

One other observation is that, in both our cases, the initial thyroid FNAC returned to be falsely reassuring, likely to be due to “sampling error” from the large thyroid mass [6]. The false negative rate is higher in thyroid nodules larger than 4 cm [7]. In such cases, a thyroid core biopsy could be a better alternative.

The presence of pituitary metastases could be challenging to manage. If the metastases are large and locally invasive, surgical clearance may be difficult. In view of close proximity to the optic chiasm as well as cranial nerves like oculomotor nerve, trochlear nerve, and abducens nerve, external beam radiotherapy and radiosurgery could be difficult to plan. In patients with poorly differentiated thyroid carcinomas, there is typically poor radioiodine uptake which would reduce the efficacy of radioactive iodine therapy. In patients in whom there is radioactive iodine uptake by the thyroid carcinoma cells, there is also a potential risk of pituitary apoplexy or haemorrhage in view of the acute increase in pituitary volume from acute swelling induced by radioactive iodine [8].

Despite aggressive therapy, prognosis for patients who have pituitary metastases from thyroid carcinoma is poor. All the patients reported in the literature passed away except one [9]. In this rare case, the 48-year-old male presented with seizures and visual disturbance and was found to have a pituitary mass. Surgical resection of the pituitary mass was performed which showed metastatic papillary thyroid carcinoma. An ultrasound thyroid was done but was normal. This patient underwent total thyroidectomy and histology showed two small foci of papillary microcarcinoma with the largest measuring 1.5 mm in greatest diameter. This patient was treated with ablative radioactive iodine (200 mci) and has been disease-free since. In contrast, most other cases fare poorer as the thyroid carcinoma foci tend to be larger and more locally advanced. Therefore, early diagnosis and collaborative management with neurosurgeons, radiation oncologists, nuclear medicine physicians, and endocrinologists is important for treatment of such patients.

## Figures and Tables

**Table 1:** Baseline laboratory results showing presence of panhypopituitarism, hyperprolactinemia, and diabetes insipidus.

Blood investigations	Result	Normal reference
Random cortisol (10 am)	**<11** nmol/L	—
Free thyroxine	**3.9** pmol/L	8.8–14.4
Thyroid stimulating hormone	0.846** **mu/L	0.65–3.70
Follicle stimulating hormone	1.7** **u/L	1.0–14.0
Luteinizing hormone	**0.1** u/L	1.0–24.0
Estradiol	38.4** **pmol/L	37.0–1284
Prolactin	**83.3** u/L	5.0–27.7
Growth hormone	0.3** **mu/L	0–28.5
Insulin-like growth factor-1	84.0** **ug/L	80–197
Sodium	147** **mmo/L	135–145
Osmolality	308** **mmol/kg	275–301
Urine osmolality	112** **mmol/kg	50–1200

**Table 2:** Summary of reported cases (including our 2 cases).

Authors	Year	Cell type^*^	Pituitary complications	Known thyroid primary	Timing from thyroid primary	RAI if known thyroid primary	Other Rx	Other sites of metastases	Age	Sex	Rx	Outcomes
Johnson and Atkins [8]	1965	P	Visual field defect CN III, CN IV	Yes	14 years	No	External beam RT	Local recurrence	56	F	Roentgen therapy Adjuvant RAI	Follow-up

Pelosi et al. [10]	1977	P	Hypopituitarism Ophthalmoplegia	No	NA	NA	No	No	32	M	Transcranial surgery	Death 1 month after presentation

Sziklas et al. [11]	1985	P	Hypopituitarism DI	Yes	25 years	No	No	Bone Chest wall	44	M	Transsphenoidal resection RAI	Death 13 months after due to massive intrathoracic haemorrhage

Masiukiewicz et al. (Case 1) [12]	1999	P	Central hypothyroidism Hypogonadism Hypoadrenalism	Yes	5 years	Yes, at diagnosis and repeated doses for recurrence	Repeated surgical clearance	Repeated local recurrence Lungs	56	M	No surgery RAI	Progressive lung and bone metastases

Masiukiewicz et al. (Case 2) [12]	1999	P	CN III deficit Hypogonadism	Yes	20 years	Yes, several years after diagnosis without clinical response	Radiosurgery	Local recurrence Bone Lungs	55	F	Stereotactic radiosurgery Surgical debulking RAI	Death after 7 months

Bell et al. [13]	2001	P	Visual field defect DI Amenorrhea	Yes	25 years	For pulmonary metastases 8 years after thyroidectomy	Neck RT at diagnosis of thyroid cancer	Lung	35	F	Transsphenoidal resection	Follow-up DI post-op

Barbaro et al. (Case 2) [14]	2013 (2011)	P	Ophthalmoplegia	Yes	2 months	Yes	No	No	65	F	Surgical intervention EBRT	Follow-up for 2 months

Trunnell and Marinelli [15]	1949	F	Visual field defect	Yes	1 year	No	No	Bone	42	F	2 RAI	Follow-up

Kistler and Pribram [16]	1975	F	Visual field defect CN III	Yes	9 years	Yes	No	No	69	F	Craniotomy but unresectable Autopsy confirming thyroid metastases	Death

Ochiai et al. [17]	1992	F	CN III, CN IV	No	NA	NA	NA	No	62	F	Transsphenoidal resection RAI	Follow-up Hormonal replacement therapy

Chrisoulidou et al. [18]	2004 (case 1996)	F	CN III	Yes	4 years	Yes	External beam RT Chemo (paraplatin/vepeside)	No	60	M	Transsphenoidal resection	Follow-up Hormonal suppressive therapy Hormonal replacement

Simon et al. [19]	2004	F	CN III, CN IV Raised ICP	No	NA	NA	NA	No	23	F	Transsphenoidal surgery abandoned RAI	Follow-up

Yilmazlar et al. [20]	2004	F	Visual field defect Raised ICP Galactorrhea	Yes	22 months	Yes	No	No	43	F	Transsphenoidal resection RAI × 3 Hormonal suppressive therapy	Follow-up

Prodam et al. [4]	2010	F	Visual disturbance Raised ICP Stalk effect Transient DI post-op	No	NA	RAI after thyroidectomy after pituitary lesion was found	No	Local lymph nodes Pelvic mets	45	F	Transsphenoidal surgery RAI	Follow-up KIV for 3rd RAI

Vianello et al. [21]	2011 (case 2001)	F	Visual field deficits Pain to right orbit Hypopituitarism	No	NA	RAI after thyroidectomy after pituitary lesion was found	External beam RT	Lung Bone Soft tissue, muscle Skull	61	F	Transnasopharyngeal biopsy Total thyroidectomy External beam radiotherapy to sellar RAI × 7	Follow-up for 10 years

Bhatoe et al. [22]	2008 (case 2001)	M	Visual field defect Stalk effect Hypogonadism-decreased libido, gynecomastia	No	NA	NA	NA	No	36	M	Craniotomy and subfrontal resection Adjuvant radiotherapy	Follow-up for 9 months

Santarpia et al. [23]	2009 (2005)	M	Raised intracranial pressure Visual field deficit DI Panhypopituitarism	Yes	15 years	No	No	Local lymph nodes Bone Lungs Liver	23	F	Transsphenoidal resection	Death 2 months after surgery due to intercurrent infection

Williams et al. [24]	2008	M	DI Visual field deficit	Yes	15 years	No	No	Lung Bone Liver Breast	23	F	Transsphenoidal resection	Follow-up

Bobinski et al. [25]	2009	M	Apoplexy Hydrocephalus Large suprasellar mass	No	NA	NA	NA	No	46	F	Craniotomy and tumour debulking	Death

Conway et al. [26]	2012	M	DI Panhypopituitarism	No	NA	NA	NA	Parotid Bilateral adrenals Bone Cerebellum	61	M	Craniotomy Palliative radiation to pituitary and combination chemotherapy	Follow-up for 13 months

Case 1	2011	A	CN III	Yes	26 days	Yes	RT	Lymph node Bone	50	F	RT RAI	Death

Case 2	2010	F	Panhypopituitarism DI Optic neuropathy	No	NA	Yes	NA	Bone Lungs	65	F	Transsphenoidal surgery RAI	Death

^*^P: papillary, F: follicular, M: medullary, and A: anaplastic.
